# Psychological Fatigue or Satisfaction? The Impact of Online Office App Use on Job Performance: Differentiating Work-Related and Non-Work-Related Contexts

**DOI:** 10.3390/bs15030283

**Published:** 2025-02-28

**Authors:** Huichuan Zhang, He Di, Mingzheng Liu, Jiaji An

**Affiliations:** 1 School of Journalism and Communication, Changchun University of Technology, Changchun 130102, China; zhanghuichuan90@163.com; 2 School of Business and Management, Jilin University, Changchun 130015, China; 3 School of Humanities, Jilin University, Changchun 130015, China; liumz@jlu.edu.cn; 4 School of Finance, Jilin University of Finance and Economics, Changchun 130117, China; 102073@jlufe.edu.cn

**Keywords:** organizational communication, online office, psychological fatigue, communication competency, workplace stress, job satisfaction, job performance

## Abstract

Media psychological fatigue, a common negative behavior among employees using online office applications (apps), has a significant impact on job satisfaction and performance. This study explores the influence of online office app use on job performance, differentiating between work-related and non-work-related contexts, based on the Uses and Gratifications Theory and the Stimulus–Organism–Response Theory. Data were collected from 418 employees in 11 enterprises in mainland China through random sampling. Harman’s single-factor test was utilized to evaluate common method bias. Regression analysis, bootstrap tests, and structural equation modeling (SEM) were used to analyze variable relationships and mediating effects. The results showed that work-related online office app use did not cause psychological fatigue or satisfaction, non-work-related app use enhanced job performance by improving job satisfaction, media psychological fatigue negatively affected job satisfaction and performance, psychological fatigue partially mediated the relationship between app use and job performance, and job satisfaction was essential for alleviating work-related fatigue and facilitating non-work-related use. This research emphasizes the dual influence of online office app use on employee perceptions and performance. Enterprises should focus on work-related app functions and foster a positive social environment with entertainment elements to encourage non-work-related use, reducing psychological fatigue and enhancing job satisfaction and performance.

## 1. Introduction

Online office applications are revolutionizing modern workplace culture. Recent data indicate that over 539 million people in China use online office software, accounting for more than 50% of all Chinese internet users. Enterprises are increasingly inclined to adopt mature or develop their own online office apps, with many leaders believing that these tools can improve mental health and prevent social media fatigue, the latter referring to the physical and mental stress caused by excessive use of personal social media (such as Facebook, Instagram, Twitter, and TikTok) during working hours (Pekkala & van Zoonen, 2022; Dantas et al., 2022). Furthermore, these applications can enhance a company’s external reputation, facilitate the development of informal internal organizations, promote knowledge transfer, and boost loyalty and job satisfaction (Nisar & Whitehead, 2016). While online office apps can substitute for personal social media to enhance work efficiency and reduce social media fatigue, questions remain: Does the proliferation of online office applications ultimately contribute to media psychological fatigue? Additionally, how do these applications impact employees’ job satisfaction and performance in work-related or non-work-related contexts? These questions form the core of our research, aiming to fill the gap in understanding the dual impact of online office apps on employee well-being and performance.

Online office applications represent an advanced work method, enabling employees to sign documents, communicate, share, and transfer files more efficiently and conveniently. In recent years, these apps have attracted the attention of communication scholars and emerged as a new frontier in management research. Previous studies have shown that online communication networks provide complementary resources (Crisp et al., 2024), enhance work performance (Okwir, 2023), moderate task characteristics (Oksa et al., 2022), and improve creativity and productivity (Pitafi, 2024). Furthermore, they enhance employees’ sense of control over their work (Soens & Claeys, 2025). Although existing research highlights the role of personal social media in promoting positive psychological impacts and work performance, most studies have focused on personal social media, with limited attention given to specialized enterprise applications. In today’s highly competitive business environment, exploring the role of online office apps is crucial for corporate management. Compared to research on personal social media, research on online office apps is still in its infancy, and further investigation into their impact on psychological fatigue and job satisfaction is needed.

To address these gaps, this study adopts the Stimulus–Organism–Response (SOR) theory as its theoretical framework. According to this theory, employees’ use of media is a proactive behavior, and their psychological state changes in response to stimuli, leading to observable reactions that can affect job satisfaction. By distinguishing between work-related and non-work-related application use, this study aims to explore how online office apps influence employees’ psychological states and job satisfaction. Data were collected through surveys from 418 employees across 11 companies in mainland China, and AMOS™ 23 and SPSS^®^ 22 (IBM Corp., Armonk, NY, USA) were used to ensure data unbiasedness and reliability through Cronbach’s alpha, construct reliability, and average variance extracted (AVE) analysis. By including media-induced psychological fatigue and job satisfaction as mediating variables, this study aimed to empirically investigate the relationships and theoretical mechanisms among these variables, providing a comprehensive understanding of how online office software use affects work performance.

The significance of this paper lies in its potential practical application value and marginal theoretical contribution. Firstly, it addresses theoretical concerns regarding the impact of online office app use. Although previous research has emphasized the role of employees’ online social behavior in promoting work performance (Ma et al., 2023), limited attention has been given to enterprise-specific applications. Secondly, by investigating media-induced psychological fatigue, job satisfaction, and work performance within the same model, this study uncovers new factors and their mechanisms, enriching the theoretical framework. Thirdly, by distinguishing between work-related and non-work-related application use, this study provides insights into how organizations can guide employees’ use of social media to enhance work performance. Overall, this study aims to provide a clear understanding of the dual impact of online office apps on employee perceptions and performance, offering valuable insights for both theory and practice.

## 2. Theoretical Analysis and Hypotheses

To establish a solid foundation for the theoretical analysis, it is crucial to clarify the core constructs adopted in this study.

Firstly, online office applications (apps) are a type of collaborative software system constructed based on cloud computing and network technologies (such as Microsoft Teams, Google Workspace, and Ding Talk). These apps are designed to provide office functions such as document processing, project management, instant messaging, and meeting collaboration via the Internet. They support real-time or asynchronous collaboration among multiple users across regions and devices (Guetz & Bidmon, 2022). The design purpose of these apps is to improve work efficiency, optimize work processes, and support the various task requirements of individuals and teams in daily work. Although online office apps are specifically designed for work purposes, with the iteration and improvement of these apps, some social attributes and functions have also been incorporated.

Secondly, referring to the research of Maseeh et al. (2024), “app use” is defined as the frequency, duration, and manner in which an individual interacts with online office apps for personal or professional purposes. This construct includes both active use (e.g., editing documents) and passive interaction (e.g., browsing files). Here, “office” encompasses all activities related to fulfilling work responsibilities within an organizational environment, including tasks such as email communication, report writing, data analysis, and attending meetings.

Thirdly, “work-related” refers to activities or behaviors that are directly related to an individual’s work responsibilities and contribute to the achievement of organizational goals. In the context of online office app use, work-related activities include using these apps to complete tasks assigned by superiors or colleagues. Conversely, “non-work-related” refers to activities or behaviors that have no direct connection with an individual’s work responsibilities. In the use of online office apps, non-work-related activities may involve personal projects, casual browsing, or social interactions unrelated to work tasks (Rugulies et al., 2023).

Fourthly, media psychological fatigue denotes the psychological state of exhaustion that individuals experience following prolonged or excessive use of media, including but not limited to social media platforms, news websites, and video streaming services. This condition is primarily characterized by a loss of interest in media content, difficulty in maintaining concentration, emotional fluctuations, and negative emotions such as anxiety and depression. It may also be accompanied by physical discomforts, such as headaches and eye strain (Dhir et al., 2018).

Katz et al. (1973) proposed the Uses and Gratifications Theory, which suggests that media users actively seek out media to fulfill specific needs. The Stimulus–Organism–Response (SOR) Theory, put forth by Mehrabian and Russell (1974), complements the Uses and Gratifications Theory by offering new insights into media psychological fatigue and job satisfaction as mediating variables. The SOR theory conceives of learning as an active process in which an organism’s psychological state responds to a stimulus and undergoes changes, leading to discernible reactions. These changes can be cognitive or emotional, whether conscious or unconscious (Lei, 2022). In this study, the use of online office apps is regarded as the stimulus (S), categorized into work-related and non-work-related uses. Media psychological fatigue and job satisfaction are considered as psychological changes in the organism (O), while job performance constitutes the response (R).

From this perspective, media fatigue stems from factors such as technical pressure (Shi et al., 2021), privacy concerns (Zhu & Bao, 2018), information overload (Cockerham et al., 2023), and social overload (An et al., 2023), which can lead to a decline in job performance. Conversely, entertainment (Zarate et al., 2022), information sharing (Bulińska-Stangrecka & Bagienska, 2021), and social interactions (Liu & Bakici, 2019) can enhance job satisfaction and modify the relationship between media use and job performance. Online office apps have alleviated media fatigue during working hours, but they have also reduced employees’ motivation to use media tools in their work (Ma et al., 2021). Furthermore, the proliferation of apps has impacted employees’ media psychological fatigue. Socializing with colleagues, venting emotions, and retrieving information are key functions of online office apps, making them appealing to employees.

### 2.1. The Relationship Between Online Office App Use and Media Psychological Fatigue

To cater to the diverse needs of employees, this study categorizes the use of online office apps into work-related and non-work-related contexts.

In work-related contexts, users often report being compelled to use online office apps for communication with colleagues. However, they have not abandoned personal social media platforms, leading to an increase in the total number of media apps used. This, in turn, heightens information exposure, communication demands, and operating system complexity. According to the SOR theory, the use of work-related online office apps, through stimulating factors such as sustained work pressure, information overload, and blurred work–life boundaries (stimulus—S), triggers psychological and physiological changes in employees, including attention fatigue, increased cognitive load, and physiological reactions (organism—O). These changes ultimately intensify media-induced psychological fatigue, manifested as decreased work efficiency, reduced job satisfaction, and physical and mental health issues (response—R). Lee et al. (2016) conducted an empirical study on the use of social network services by Korean employees and found that information overload, communication overload, and system feature overload can lead to physical and psychological stress. S. Zhang et al. (2016) further proposed that frequent system notifications, such as message reminders, can elicit irritability, fatigue, and dissatisfaction.

In non-work-related contexts, excessive and frequent online socializing or entertainment activities can also evoke negative emotional responses in users. Based on the SOR theory, the use of non-work-related online office apps, through their attractiveness and distractive nature (S), acts on employees’ psychological fatigue state (O), triggering negative emotions, decreased attention, and reduced work efficiency (R). This stimulus–organism–response cycle further reinforces media-induced psychological fatigue. Islam et al. (2020) emphasized that multiple motives for social media use can lead to problematic consequences, especially for those who are more inclined to share unverified information for self-promotion or entertainment purposes, as they are more likely to experience social media fatigue. Zhu and Bao (2018) also pointed out that excessive social information and inappropriate privacy monitoring can trigger media fatigue. Media-induced psychological fatigue is characterized by a subjective experience that encompasses a variety of negative emotions, such as boredom, burnout, disappointment, and indifference (Ravindran et al., 2013).

Based on the foregoing theoretical analysis, we propose the following hypotheses:

**H1:**  *Work-related online office app use has a significant positive impact on media psychological fatigue.*

**H2:**  *Non-work-related online office app use has a significant positive impact on media psychological fatigue.*

### 2.2. The Relationship Between Online Office App Use and Job Satisfaction

The relationship between online office app use and job satisfaction is complex. In work-related contexts, two contrasting viewpoints have emerged. Grelle and Popp (2021) observed that telecommuting can exacerbate feelings of isolation, thereby diminishing job satisfaction and performance. Analogously, Yin et al. (2018) found that employees compelled to use enterprise social media or other information and communication technologies experience a markedly lower level of job satisfaction. Conversely, Zürcher et al. (2021) reported that the increase in job satisfaction during remote work was not attributable to work–life balance but rather to a reduction in unnecessary interruptions. On the one hand, online office apps serve as specific software tools; on the other hand, their utilization in online work environments can enhance employee focus and enthusiasm (Strunk & Strich, 2023). When comparing the negative and positive impacts, the latter appears to be more prominent and significant. Under the framework of the SOR theory, the first view does not fully consider the collaborative and social support functions of online office apps (S), as well as employees’ adaptability to and demand for these tools (O). In contrast, the second perspective reflects communication and collaboration, as well as personalization and flexibility (S), which affect employees’ work needs and expectations and psychological state (O), and trigger improved work efficiency, reduced stress, and enhanced autonomy and sense of control (R). Therefore, this paper adopts the second viewpoint as the research hypothesis, namely H3.

The use of non-work-related online office apps triggers positive emotional experiences, enhanced sense of belonging and team cohesion, and the fulfillment of individualized needs among employees through stimulating factors such as providing entertainment and relaxation, facilitating social interaction, and meeting personalized demands. These psychological and physiological responses ultimately lead to a significant increase in job satisfaction. Jo and Baek (2023) found that employees employ social networking services to relieve stress during work hours, contributing to relaxation. König and Delaguardia (2014) demonstrated that employees who engage more with friends and family via social networking services during work hours report a higher level of job satisfaction. Bulińska-Stangrecka and Bagienska (2021) further noted that online office app users develop closer relationships due to the connectivity enabled by the software, which augments trust and contributes to greater job satisfaction.

Based on the above theoretical analysis, this study proposes the following hypotheses:

**H3:**  *Work-related online office app use has a significant positive influence on job satisfaction.*

**H4:**  *Non-work-related online office app use has a significant positive influence on job satisfaction.*

### 2.3. The Relationship Between Media Psychological Fatigue and Job Satisfaction

Based on the SOR theory, media psychological fatigue arises from stimulating factors such as information overload, continuous attention demands, and blurred boundaries between work and life, triggering psychological and physiological responses in employees, including attention fatigue, emotional low, and physical exhaustion (Shokouyar et al., 2018). In the context of online office app use, internal psychological alterations manifest as negative emotions, while external behaviors may encompass disobedience, delayed responses, prolonged absences, or resignation. Hwang et al. (2020) concluded that employees’ online social anxiety, cognitive fatigue, and perceived information overload due to instant messaging negatively affect work engagement. Empirical research suggests that information overload augments online social anxiety and diminishes job satisfaction and engagement. Therefore, this paper hypothesizes that media psychological fatigue induced by online office app use negatively impacts job satisfaction.

Based on the above theoretical analysis, this study proposes the following hypothesis:

**H5:**  *Media psychological fatigue has a significant negative influence on job satisfaction.*

### 2.4. The Relationship Between Media Psychological Fatigue and Job Performance

With the advent of 5G networks, enterprises are increasingly relying on enterprise network service platforms to facilitate effective teamwork, knowledge coordination, and knowledge creation, thereby enhancing group performance (Cao & Ali, 2018). However, excessive media exposure can overwhelm users, generating negative emotions such as fatigue, irritability, apathy, and disgust, which curtail employee motivation and innovation (Good et al., 2016; Imas et al., 2017; Labban & Bizzi, 2022). Given the similarity in the impact of media-induced psychological fatigue on job satisfaction and job performance within the same theoretical framework, it can be inferred from the preceding discussion that media psychological fatigue has a negative effect on job performance.

**H6:**  *Media psychological fatigue has a significant negative influence on job performance.*

### 2.5. The Relationship Between Job Satisfaction and Job Performance

Through the framework of the SOR theory, job satisfaction acts as a stimulus that influences the organismic state of employees, thereby triggering positive work behaviors and responses and ultimately exerting a significant positive impact on job performance. Bakotić (2016) explored the link between job satisfaction and organizational performance, demonstrating a bidirectional relationship where job satisfaction drives organizational performance. Chi et al. (2023) examined the moderating effects of financial and non-financial rewards on the relationship between job satisfaction and job performance, finding a robust association between the two. Wong et al. (2021) analyzed responses from 758 hotel employees in the United States using *t*-tests and structural equation modeling, revealing that job satisfaction and organizational commitment significantly account for job performance, subjective well-being, and prosocial behavior.

Based on the above theoretical analysis, this study proposes the following hypothesis:

**H7:**  *Job satisfaction has a significant positive influence on job performance.*

**Figure 1:**
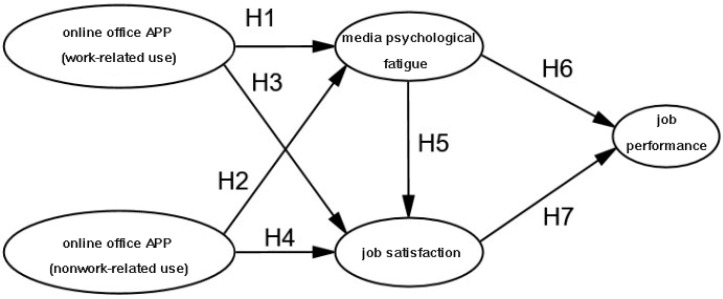
Structural equation modeling of study hypotheses.

In summary, this study constructs a structural equation model based on the theoretical analysis and proposed hypotheses (Figure 1).

## 3. Materials and Methods

### 3.1. Questionnaire Design and Variable Definition

The questionnaire employed in this study consisted of two sections: personnel information and measurement questions. All measurement items were structured as declarative statements, encompassing control variables such as gender, age, educational background, length of service, duration of online office app usage, and daily usage time. The measurement variables were rated on a 7-point Likert scale, ranging from 1 (very inconsistent) to 7 (very consistent). To ensure validity and scientific rigor, the original measurement variables utilized in this study were derived from the established literature.

The independent variable, namely online office app usage, was assessed using a well-validated questionnaire developed by Katsarou et al. (2022), tailored to the context of online office apps. This scale encompassed nine items, divided into five work-related use items and four non-work-related use items. The media psychological fatigue scale was adapted from the study by S. Zhang et al. (2016), consisting of four items. The job satisfaction scale was modified from Toropova et al.’s (2021) research, aligned with a six-dimensional model more suited to Chinese contexts, comprising six items. The job performance scale integrated insights from Darvishmotevali and Ali (2020), with slight adjustments to reflect Chinese realities, and included six items. The variable definitions are presented in Table 1.

Initially, the questionnaire comprised 31 questions, including 6 informational items and 25 variable-related items.

### 3.2. Data Sources

This study utilized the Wenjuanxing platform, a prominent questionnaire tool in China, leveraging university alumni associations and graduates to conduct an online survey. A total of 442 employees from 11 enterprises across various sectors, including banking, media, academia, finance, automotive, healthcare, and advertising, participated. Prior to widespread distribution, a pilot survey involving 40 participants was conducted to identify and rectify potential issues, such as unclear wording, obscure terminology, and ambiguities.

Based on the pilot survey feedback and expert advice from management and communication specialists, the initial questionnaire underwent revisions. The final sample was balanced in terms of gender, with the majority of participants aged 30–39 years (34.7%) and 80% under the age of 50. Most participants had a Master’s degree or higher (55.5%), with 93.3% having at least one year of service and 82% using online office apps for more than two hours daily. Consequently, the sample data demonstrated good representativeness and reliability for this study. Table 2 presents the basic demographic information of the sample.

To analyze the possibility of sociodemographic differences, this study conducted a regression analysis on the basic characteristics of the respondents and their usage of online office apps. Due to the high correlation between age and length of service, we aimed to avoid multicollinearity among the independent variables. As a result, we excluded the age variable, which contained less information. Other variables such as gender, education, and length of service were selected for inclusion in the regression model. There are two dependent variables, namely time of online office app use and daily online office app use time, which are included in Model A and Model B, respectively. The results of the regression analysis are shown in Table 3. From Model A, it can be seen that an individual’s length of service is significantly positively correlated with the time of online office app use, which seems to be a given and will not be discussed further. Factors such as gender and education level do not have a significant impact on the dependent variable in Model A. Based on the data from Model B, we obtained interesting findings: employees with higher education levels tend to use online office apps more frequently, and female respondents may also use them more often. This may suggest that female employees with higher education levels are likely to use online office apps more frequently.

### 3.3. Reliability Analysis and Validity Test

In this study, both SPSS^®^ 22 and AMOS™ 23 were utilized to assess the reliability and validity of the sample data by examining Cronbach’s alpha, composite reliability, and discriminant validity. Initially, composite reliability and convergence were evaluated. The results revealed that the standardized loadings of the measurement items were all above 0.6, the construct reliability values for all variables exceeded 0.8, and Cronbach’s alpha coefficients were all greater than or nearly equal to 0.8. These findings indicate that the data collected via the questionnaire were reliable and satisfied the measurement criteria. Furthermore, the AVE (average variance extracted) values for all variables were greater than 0.5, suggesting the high convergent validity of the questionnaire data. The specific data and questionnaire items are presented in Table 4.

To assess discriminant validity, the square root of the AVE for each variable was calculated. The diagonal values in Table 5 represent the square roots of the AVEs, and each of these values is greater than the other correlation coefficients in the corresponding row and column. This indicates that the scale items were designed with a high level of differentiation, supporting subsequent analyses.

To mitigate the risk of common method bias in the data, this study adopted the test proposed by Podsakoff et al. (2003). During the data compilation phase, a manual review was conducted to scrutinize the data. Questionnaires with identical IP addresses and those with uniformly identical answers were excluded. Following data collection and compilation, we analyzed the variance explained using Harman’s single-factor test in SPSS^®^ 22. The results showed that five principal components were extracted from the sample, with the first common factor explaining the largest percentage of variance (31.247%). Since this value was below the threshold of 40%, as recommended in the literature, we concluded that the data were not affected by common method bias.

## 4. Results

### 4.1. Model Fit Verification

**Figure 2:**
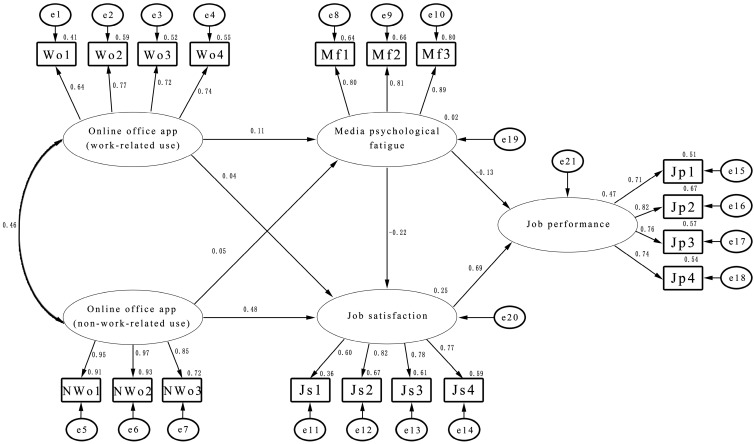
Structural equation model of online office apps and job performance.

Based on theoretical analysis, we established a structural equation model (SEM), where the variables involved were self-reported by employees. Given this, the fit of the SEM needed to be examined before proceeding with subsequent analyses. The fit of the SEM was assessed using AMOS™ 23, with parameters estimated using maximum likelihood. The results of the SEM analysis, based on the sample data obtained from the research, are presented in Figure 2. It was observed that all fit indicators met the statistical criteria for SEM (x^2^ = 410.882; df = 127; x^2^/df = 3.235; RMSEA = 0.073; GFI = 0.899; CFI = 0.934; AGFI = 0.864; PGFI = 0.668). Therefore, the model demonstrated a good fit with the prior theoretical analysis and provided initial support for subsequent hypothesis testing.

### 4.2. Hypothesis Testing

The sample data from the questionnaires were integrated into the structural equation model to test hypotheses H1–H7. The results indicated that neither work-related nor non-work-related online office app use significantly affected media psychological fatigue, thus failing to support H1 and H2. Work-related online office app use did not significantly influence job satisfaction, whereas non-work-related use had a significant positive effect on job satisfaction, supporting H4 but not H3. Media psychological fatigue had a significant negative effect on job satisfaction, suggesting that higher levels of media psychological fatigue among employees corresponded to lower levels of job satisfaction, supporting H5. Additionally, media psychological fatigue had a significant negative effect on job performance, while job satisfaction had a significant positive effect on job performance, supporting both H6 and H7. The detailed results are presented in Table 6.

### 4.3. Analysis of Mediating Effects

For the examination of mediating effects, this research employed the bootstrapping algorithm with 2000 replicated sample draws. Specifically, we examined the mediating effect of job satisfaction on the relationship between media psychological fatigue and job performance using bootstrap analysis. Three models were established for this purpose: Model A, where the independent variable was directly related to the dependent variable; Model B, where the independent variable was related to the dependent variable only through the mediating variable without a direct connection; and Model C, where the independent variable was directly related to the dependent variable and also related to the dependent variable through the mediating variable.

We tested the relationship between non-work-related use of online office apps and job performance, with job satisfaction acting as a mediating variable. In Model A, the relationship was statistically significant. In Model C, the statistical significance of the relationship disappeared, but the effect of job satisfaction as a mediating factor on job performance remained statistically significant. In Model B, the effect of non-work-related use of online office apps on job satisfaction was still statistically significant. Therefore, job satisfaction exhibited a complete mediating effect between non-work-related use of online office apps and job performance. Using this method, we tested the mediated paths one by one. In other paths, the effects of independent variables on the dependent variable were statistically significant in Model C, indicating that the mediating effects were incomplete. The detailed results are shown in Table 7.

## 5. Conclusions and Discussion

### 5.1. Conclusions

This paper delves into the intricacies of the relationships among online office app usage, media psychological fatigue, job satisfaction, and job performance, grounded in the Uses and Gratifications Theory and the SOR Theory. It also presents a set of corresponding hypotheses. Through an empirical investigation involving 418 employees from Chinese enterprises, we conducted rigorous testing and analyzed the mediating effects utilizing the bootstrap method. The salient findings are as follows.

Firstly, our empirical results indicated that, irrespective of whether online office app usage during work hours was work-related or non-work-related, there was no statistically significant effect on media psychological fatigue. Although the data did not support hypothesis H1, they approached the statistical threshold (*p* = 0.05), suggesting a potential for work-related online office app use to induce media psychological fatigue in real-world contexts. Upon scrutinizing the survey data, two plausible explanations for the unsupported hypothesis emerged: On the one hand, the daily usage of online office apps was less than four hours, allowing employees to manage the information load effectively. Conversely, prolonged use exceeding four hours could lead to media information overload and social overload, potentially triggering fatigue. The other reason could be the high media literacy of the surveyed employees, who were predominantly from functional departments with strong educational backgrounds, which might have conferred resistance to fatigue. This correlation between education and media resilience echoes Lee et al.’s (2016) study on SNSs in South Korea, which highlighted the stress induced by information and communication overload.

Secondly, work-related online office app usage exhibited no significant impact on job satisfaction, whereas non-work-related usage had a notably positive effect. This finding resonates with Gonzalez et al.’s (2013) research on social media, which found no significant tie between work-related social media use and organizational commitment but noted that social-oriented use could stabilize and clarify organizational culture. Analyzing these results in tandem suggests that work-related media use functions more as a hygiene factor, while non-work-related use, encompassing entertainment and socialization, acts as a motivator. Thus, non-work-related media use fosters employee satisfaction, while work-related use merely avoids dissatisfaction, aligning with Herzberg’s theory from 1966.

Thirdly, media psychological fatigue exhibited a significant negative correlation with both job satisfaction and job performance. Conversely, job satisfaction and job performance were positively and significantly related. This implies that employee media psychological fatigue detracts from job performance, but heightened job satisfaction can mitigate this effect. The detrimental impact of media psychological fatigue on work enthusiasm, satisfaction, and performance aligns with broader research on media fatigue (Labban & Bizzi, 2021; Kasim et al., 2022). Only judicious media use positively influences job performance through job satisfaction (X. Zhang et al., 2019).

Lastly, the bootstrapping test results revealed that job satisfaction fully mediated the effect of non-work-related online office app use on job performance, contrasting with its incomplete mediation of work-related use’s impact. This disparity may stem from employees’ perception of online office apps primarily as work tools, with non-work-related functions viewed as unexpected benefits. The distinct mediating effects of work-related and non-work-related use highlight this perception. Media psychological fatigue incompletely mediated the relationship between non-work-related use and job performance, possibly due to the multifaceted impacts of activities like communication, socializing, and entertainment on informal organizational dynamics. The incomplete mediation of job satisfaction between media psychological fatigue and job performance may reflect job satisfaction’s complexity as a comprehensive feeling influenced by factors such as life satisfaction (S. X. Zhang et al., 2021) and perceived organizational politics (Abbas et al., 2014).

### 5.2. Contribution

Our research is innovative and has the potential to make both theoretical and practical contributions.

Firstly, we highlight the focus on online office app use by distinguishing between work-related and non-work-related situations. Although existing research has explored the role of social networking sites (SNSs) in affecting employee job performance, most studies have primarily focused on social media without distinguishing the purpose of use. In this paper, we attempt to investigate how online office app use affects job performance. On one hand, our findings extend the theoretical research on network services and job performance. On the other hand, they enrich the research by X. Zhang et al. (2019), which reported not only that personal private social media use affects job satisfaction and turnover intention but also that using online office apps can influence the media psychological fatigue, job satisfaction, and job performance of employees.

Secondly, this study proposes a new logical path for research. Based on the Uses and Gratifications Theory and the Stimulus–Organism–Response (SOR) Theory, we innovatively distinguish media use into work-related and non-work-related types, explaining why there are often two opposite conclusions regarding media use during work time. There is a limited body of literature focusing on online office apps, especially the different requirements of use, and our research may emphasize the importance of media use needs.

Thirdly, some new practical insights can be drawn from the empirical findings of this study. Enterprises should develop more non-work-related modules and functions for online office apps to create additional use cases beyond work use. These non-work-related modules, such as socializing and entertainment, can help establish informal organizations, promote efficient knowledge transfer, increase employee job satisfaction, and thereby enhance organizational commitment and job performance, ultimately reforming the idea among employees that online office apps consume their energy and resources (Zivnuska et al., 2019). At the same time, it is crucial to supervise and manage the time employees spend on online office apps, setting different use times and total amounts of information for different roles. Employees should strive to use media for work no more than 4 h per day to avoid media psychological fatigue, which can influence job performance. Additionally, employees should be allowed to set personalized reminder tones for online office apps to avoid triggering media psychological fatigue, as reminder alerts are an important triggering factor for the perception of information overload (S. Zhang et al., 2016).

### 5.3. Limitations and Prospects

There are also limitations in our study. We only considered the mediating role of media psychological fatigue and job satisfaction in the relationship between online office app use and job performance from the perspective of the SOR model. However, there may be other mediating paths. Future research could explore the role of additional mediating variables, such as work engagement, organizational commitment, and technostress, in the relationship from emotional and cognitive perspectives. This study is based on data from enterprises in China due to data availability. However, there are inconsistencies in attitudes toward media, media exposure, and media culture between China and Western countries. As the global information infrastructure is constructed step by step, working online with apps is becoming increasingly common. Therefore, it is important to explore the deep connotations and influence of online office app use. This is key to revealing employee behaviors and helping enterprises improve efficiency, reduce costs, and successfully transform in the information era.

## Figures and Tables

**Table 1:** Core variable definitions.

Core Variable	Abbreviation	Conceptual Description
Online office app usage	Wo	Duration and frequency of work-related online office app usage.
	NWo	Duration and frequency of non-work-related online office app usage.
Media psychological fatigue	Mf	Psychological state of exhaustion that individuals experience following prolonged or excessive use of media.
Job satisfaction	Js	The degree of employees’ psychological and emotional recognition and pleasure in work.
Job performance	Jp	Employees’ performance and achievements in completing tasks, achieving goals, fulfilling responsibilities, and other aspects at work.

**Table 2:** Basic information of the data sample (N = 418).

Questions	Items	Frequency	Percentage
Gender	Male	196	46.9
	Female	222	53.1
Age	20–29	138	33.0
	30–39	145	34.7
	40–49	53	12.7
	50–59	73	17.5
	Over 60	9	2.2
Education	College or below	28	6.7
	Bachelor’s	158	37.8
	Master’s or above	232	55.5
Length of service	<1 year	26	6.2
	1–3 years	79	18.9
	3–5 years	113	27.0
	>5 years	200	47.8
Time of online office app use	Less than 1 year	8	1.9
	1–2 years	57	13.6
	3–5 years	95	22.7
	>5 years	258	61.7
Daily online office app use time	<1 h	16	3.8
	1–2 h	59	14.1
	2–4 h	184	44.0
	>4 h	159	38.0

**Table 3:** Results of regression analysis based on sociodemographic characteristics.

Independent Variable	Model A Time of Online Office App Use				Model B Daily Online Office App Use Time			
	β	*t*-Value	*p*-Value	VIF	β	*t*-Value	*p*-Value	VIF
Gender	0.002	0.052	0.959	1.003	0.266 ***	6.097	0.000	1.003
Education	−0.038	−0.842	0.400	1.010	0.358 ***	8.165	0.000	1.010
Length of service	0.412 ***	9.146	0.000	1.007	0.043	0.977	0.329	1.007
R^2^	0.318				0.362			

Notes: *** represents *p* < 0.001.

**Table 4:** Reliability and validity tests of questionnaire items (N = 418).

Questions	Items	Std.	CR	AVE	α
Wo	W1: I always have meetings on online office apps with colleagues.	0.614	0.809	0.517	0.799
	W2: I use online office apps to share policy and information with colleagues.	0.781			
	W3: I use online office apps to upload, download, and transfer files.	0.733			
	W4: I use online office apps to find colleagues with specific knowledge or expertise.	0.737			
NWo	NW1: I use online office apps to communicate with colleagues after work.	0.952	0.945	0.853	0.944
	NW2: I use online office apps to make friends within the enterprise.	0.966			
	NW3: I use online office apps to entertain myself and relax during free time at work.	0.848			
Mf	F1: Using online office apps can be boring and uninteresting to me sometimes.	0.799	0.875	0.700	0.873
	F2: Compulsory online office app use makes me tired.	0.817			
	F3: I feel exhausted and lost after using online office apps sometimes.	0.891			
Js	S1: I can get a nice financial reward from my job.	0.622	0.836	0.564	0.831
	S2: My job makes me feel pleasure and I like what I do.	0.823			
	S3: There are enough opportunities for advancement in my job and it is fair.	0.816			
	S4: I enjoy working with my colleagues and superiors, who are competent in their roles.	0.724			
Jp	P1: I have the knowledge and skills to complete the work I need, and I can finish my work on time.	0.724	0.843	0.573	0.839
	P2: I am glad to give a hand when my colleagues are asking for help.	0.828			
	P3: I am able to get help from colleagues or superiors when I am in trouble.	0.729			
	P4: I enjoy improving myself and learning new knowledge and skills at work.	0.743			

**Table 5:** Discriminant validity test.

Variable	Jp	Js	Mf	NWo	NWo
Jp	0.757				
Js	0.672	0.751			
Mf	0.039	0.099	0.837		
NWo	0.251	0.396	0.013	0.924	
Wo	0.478	0.476	0.101	0.488	0.719

**Table 6:** Results of hypothesis testing.

Hypothesis	Unstd.	S.E.	C.R.	P	Result
Wo→Mf (H1)	0.111	0.059	1.881	0.057	No
NWo→Mf (H2)	0.063	0.077	0.858	0.414	No
Wo→Js (H3)	0.025	0.036	0.694	0.448	No
NWo→Js (H4)	0.357	0.057	6.270	***	Yes
Mf→Js (H5)	−0.329	0.034	−9.676	***	Yes
Mf→Jp (H6)	−0.114	0.022	−5.182	***	Yes
Js→Jp (H7)	0.507	0.055	9.212	***	Yes

Notes: *** represents *p* < 0.001.

**Table 7:** Results of mediation analysis.

Path			Model A	Model B	Model C		Result
Independent Variable (Iv)	Mediator Variable (Mv)	Dependent Variable (Dv)	Iv-Dv	Iv-Mv	Iv-Dv	Mv-Dv	
Wo	Mf	Jp	0.48 ***	0.11			None
NWo	Mf	Jp	0.55 ***	0.14 ***	0.35 ***	0.52 ***	Incomplete
Wo	Js	Jp	0.48 ***	0.50 ***	0.22 ***	0.57 ***	Incomplete
NWo	Js	Jp	0.55 ***	0.26 ***	0.02	0.67 ***	Complete
Mf	Js	Jp	−0.23 ***	−0.20 ***	−0.13 ***	0.62 ***	Incomplete

Notes: *** represents *p* < 0.001.

## Data Availability

The data that support the findings of this study are available from the corresponding author, He Di, upon reasonable request.
